# Supplementary material for: Effect of different forage-to-concentrate ratios on ruminal bacterial structure and real-time methane production in sheep
Source: PLoS One. 2019 May 22;14(5):e0214777. doi: 10.1371/journal.pone.0214777 (PMC6530836; doi:10.1371/journal.pone.0214777)
Supplement: S1 Dataset — (DOCX) [file pone.0214777.s001.docx]

| **Group** | **Copy** | **AA** | **PA** | **BA** | **pH** | **AA/PA** | **NH_3_-N** | **IVDMD** |
| --- | --- | --- | --- | --- | --- | --- | --- | --- |
| **Unit** | **-** | **mmol/L** | **mmol/L** | **mmol/L** | **-** | **-** | **mg/100mL** | **%** |
| L | 1 | 44.71 | 12.18 | 8.64 | 6.43 | 3.67 | 32.08 | 55.65 |
| L | 2 | 45.06 | 13.59 | 8.05 | 6.99 | 3.32 | 26.98 | 58.40 |
| L | 3 | 45.77 | 13.90 | 8.64 | 6.41 | 3.29 | 28.50 | 58.85 |
| L | 4 | 46.69 | 15.57 | 8.38 | 6.99 | 3.00 | 26.95 | 60.20 |
| L | 5 | 47.97 | 18.41 | 7.98 | 6.31 | 2.61 | 31.53 | 64.47 |
| L | 6 | 47.97 | 18.90 | 9.17 | 6.60 | 2.54 | 27.17 | 66.20 |
| L | 7 | 50.59 | 18.99 | 9.49 | 6.88 | 2.66 | 28.88 | 68.59 |
| L | 8 | 50.86 | 19.19 | 8.19 | 6.47 | 2.65 | 31.35 | 69.57 |
| L | 9 | 52.17 | 19.59 | 9.23 | 6.85 | 2.66 | 32.13 | 70.97 |
| M | 1 | 47.13 | 11.86 | 8.03 | 6.42 | 3.97 | 31.41 | 56.57 |
| M | 2 | 47.24 | 11.95 | 7.53 | 7.20 | 3.95 | 26.57 | 59.33 |
| M | 3 | 48.20 | 12.86 | 8.03 | 6.60 | 3.75 | 28.02 | 60.03 |
| M | 4 | 48.49 | 14.58 | 7.81 | 7.20 | 3.33 | 26.54 | 60.69 |
| M | 5 | 49.53 | 15.66 | 7.47 | 6.50 | 3.16 | 30.88 | 62.29 |
| M | 6 | 49.53 | 16.05 | 8.49 | 6.80 | 3.09 | 26.76 | 62.44 |
| M | 7 | 51.55 | 16.25 | 8.76 | 7.09 | 3.17 | 28.37 | 65.30 |
| M | 8 | 51.92 | 16.50 | 7.65 | 6.67 | 3.15 | 30.72 | 65.44 |
| M | 9 | 52.27 | 17.02 | 8.53 | 7.06 | 3.07 | 31.45 | 67.37 |
| H | 1 | 46.24 | 10.65 | 8.33 | 6.73 | 4.34 | 28.84 | 52.95 |
| H | 2 | 48.62 | 10.85 | 7.14 | 7.32 | 4.48 | 24.80 | 54.94 |
| H | 3 | 49.40 | 10.97 | 8.43 | 6.71 | 4.50 | 26.01 | 57.20 |
| H | 4 | 50.41 | 11.36 | 7.42 | 7.32 | 4.44 | 24.78 | 57.65 |
| H | 5 | 51.81 | 12.03 | 6.98 | 6.41 | 4.31 | 28.40 | 60.10 |
| H | 6 | 51.81 | 14.51 | 8.68 | 6.91 | 3.57 | 24.96 | 64.67 |
| H | 7 | 54.67 | 15.07 | 8.75 | 7.20 | 3.63 | 26.31 | 66.60 |
| H | 8 | 54.97 | 15.86 | 7.06 | 6.78 | 3.47 | 28.26 | 67.59 |
| H | 9 | 57.40 | 16.69 | 8.73 | 7.18 | 3.44 | 28.87 | 71.91 |
