# Supplementary material for: Effect of different forage-to-concentrate ratios on ruminal bacterial structure and real-time methane production in sheep
Source: PLoS One. 2019 May 22;14(5):e0214777. doi: 10.1371/journal.pone.0214777 (PMC6530836; doi:10.1371/journal.pone.0214777)
Supplement: S2 Dataset — (DOCX) [file pone.0214777.s002.docx]

| **Group** | **Copy** | **General bacterial** | **Protozoa** | **Methanogens** | **Anaerobic fungi** |
| --- | --- | --- | --- | --- | --- |
| Unit | - | % | % | ‰ | ‰ |
| L | 1 | 112.92 | 4.87 | 6.00 | 1.71 |
| L | 2 | 101.24 | 4.03 | 5.12 | 1.29 |
| L | 3 | 110.99 | 4.52 | 5.63 | 1.82 |
| L | 4 | 98.39 | 4.14 | 5.23 | 1.52 |
| L | 5 | 120.36 | 4.49 | 5.60 | 1.61 |
| L | 6 | 103.69 | 4.15 | 5.25 | 1.52 |
| M | 1 | 80.45 | 3.78 | 4.86 | 1.35 |
| M | 2 | 97.56 | 4.12 | 5.26 | 1.68 |
| M | 3 | 109.41 | 4.21 | 5.37 | 1.92 |
| M | 4 | 102.67 | 4.37 | 5.55 | 1.78 |
| M | 5 | 104.28 | 4.44 | 5.65 | 1.81 |
| M | 6 | 97.32 | 4.11 | 5.25 | 1.68 |
| H | 1 | 98.17 | 4.54 | 5.33 | 1.92 |
| H | 2 | 81.05 | 3.62 | 4.92 | 1.76 |
| H | 3 | 97.14 | 4.42 | 5.56 | 2.01 |
| H | 4 | 94.79 | 4.09 | 5.14 | 1.44 |
| H | 5 | 78.03 | 4.01 | 5.04 | 2.20 |
| H | 6 | 95.41 | 4.12 | 5.17 | 1.86 |
